# Waste of Horse Life

**Published:** 1881-02

**Authors:** 


### WASTE OF HORSE LIFE.

The number of horses in the United Kingdom has been estimated at rather more than 2,250,000, and their average value can scarcely be set down at less than £30. Their collective value, therefore, falls little short of £28,000,000. That the nation incurs a loss if this sum is spent quicker than it needs to be is a self-evident proposition; that it is so spent is certain, if horses on an average become useless at a time when they ought still to be in full vigor. On this point few will be disposed to challenge the verdict of Mr. W. Douglas, late veterinary surgeon in the Tenth Hussars, who tells us that a horse should live from 35 to 40 years, and live actively and usefully three-fourths of this period. "All authorities," he says, "now admit that animals should live five times as long as it takes them to reach maturity. A dog, which is at its full growth when between 2 and 3 years old, is very aged at 12 years. Horses do not, unless their growth is forced, reach their prime until they are 7 or 8 years old, which, by the same law, leaves them to live some 30 years longer. When these facts are kept in mind, together with the other facts that three-fourths of our horses die or are destroyed under 12 years old, that horses are termed aged at 6," (he should have said 8) "old at 10, very old when double that number of years, and that few of them but are laid up from work a dozen times a year, * * * the viciousness of a system which entails such misery and destruction of life cannot be too strongly commented upon." If we take the age of 3 years as that at which horses begin to work, and 12 as that at which they are worn out, it follows that the period of their efficiency is shorter by at least 14 years than it should be. In other words, the nation has to buy three horses when it ought to buy only one, and thus upward of £200,000,000 are spent every 21 years in the purchase of horses, when £68,000,000 ought to suffice. The loss, therefore, to the nation at least is $135,000,000 in 21 years.—*Sir George W. Cox, in Fraser's Magazine.*

---

A LITTLE four-year-old was at one of our photograph studios having her picture taken. The artist said: "You must keep your mouth shut, my dear, and your eyes open," as the little miss showed a decided inclination to open her mouth and close her eyes. But on being instructed she braced up, and after a few minutes wonderingly asked: "Now, what shall I do with my nose?"

### MORAL.

If farmers as a class will not take an interest in public affairs, they may expect to be preyed upon by the railroads. Railroad men now boldly advocate a new principle of fixing charges—viz., "what the traffic will bear," instead of the old one upon which carriers' charges were based—"cost of service." Once recognize the new theory—with watered stock for an excuse and the new pooling system as the power—the productions of a continent are at the mercy of the corporations which the people have created. In order to perpetuate this system, railroad managers naturally seek political power. Both the chairmen of the Democratic and Republican national committees at this time are railroad men, and railroad money is largely relied upon to run the political machinery. Congress, and also many of our State Legislatures, are controlled by the railroads. First, through officers or directors who become legislators for that purpose ; second, and perhaps more largely, through members of the legal profession who are also legislators, and are retained as "counsel" by the railroads ; third, through special favors shown all the members.

Is it not about time that the farmers, who constitute by far the largest single interest in the country, and whose productions are the basis of all our prosperity, should have something to say about the amount they are taxed for transportation ? In England the Farmers' Alliance held the balance of power at the last general election. What is to prevent their organizing in every assembly and congressional district here and takeing an interest in politics which would not only protect their own interest, but be an efficient check upon the encroachments of our corporations, which have been so great of late that all patriotic citizens must view their increasing power with alarm.—*Graphic.*

---

Crossness and Irritability.—Much of the crossness, irritability and general unamiableness which characterize certain children and make their presence so annoying springs from neglect of their happiness in some direction. Either from indiscreet indulgence, undue severity, or careless negligence, their physical system is out of order, or their tempers are soured, and, feeling uncomfortable, they naturally vent their discomfort upon others. In describing a young child the words "good" and "happy" are almost synonymous, and no effort to make him the former can be successful as long as the latter is neglected.

### THE WOODEN MAN.

The most interesting statue to us, and perhaps the oldest image in Egypt, and, if so, in the world, is the Wooden Man, which was found at Memphis. This image, one meter and ten centimeters high, stands erect, holding a staff. The figure is full of life, the *pose* expresses vigor, action, pride; the head, round in form, indicates intellect. The eyes are crystal, in a setting of bronze, giving a startling look of life to the regard. It is no doubt, a portrait. "There is nothing more striking," says its discoverer, "than this image, in a manner living, of a person who has been dead six thousand years." He must have been a man of mark, and a citizen of a State well civilized; this is not a portrait of a barbarian, nor was it carved by a rude artist. Few artists, I think, have lived since who could impart more vitality to wood. And if the date assigned to this statue is correct, sculpture in Egypt attained its maximum of development six thousand years ago.

---

A Paper House and Paper Furniture.—The multiplying applications of paper are strikingly illustrated by the fact that in the recent Sydney exhibition there was shown a house, built and furnished throughout with articles made of paper. The structure was one story high, with skeleton or frame-work made of wood. The exterior was covered with panels made of paper pulp, while the intertor was covered with the same material—plain on the floor, but forming splendid arabesques on the walls, and molked in imitation of plaster on the ceilings. The doors, cupboards and shelves were of the same material, while the entire furniture, including chandeliers and a stove in which a fire could be lighted, was made of paper-maché. The carpets and curtains were of paper, and there was a bed-room, in which there was not only a large bedstead made of papier-maché, but blankets, sheets, quilts, and female under-clothing, dresses and bonnets in the latest style, composed solely of carton-pâte. A series of banquets were given in this building, in which plates, dishes, knives, forks and glasses were all of paper.

---

It is unnecessary to debate so obvious a question as to which side a man and woman should respectively take. It is always right for the gentleman to take the left, and it is then left for the lady to take the right.

